# The Role of the Second Coordination Sphere in the Biological Activity of Arene Ruthenium Metalla-Assemblies

**DOI:** 10.3389/fchem.2018.00602

**Published:** 2018-12-11

**Authors:** Bruno Therrien

**Affiliations:** Institute of Chemistry, University of Neuchatel, Neuchatel, Switzerland

**Keywords:** arene ruthenium, metalla-assemblies, bio-inorganic, piano-stool complexes, weak interactions, second coordination sphere

## Abstract

For nearly 15 years, the biological and biomedical applications of arene ruthenium metalla-assemblies have flourished. Today, the synthetic strategies to generate arene ruthenium assemblies are well-established, and these compounds offer tremendous possibilities in terms of structural diversities and chemical properties. However, the second coordination sphere is often poorly considered, if not ignored, when designing such arene ruthenium metalla-assemblies. These weak interactions (hydrogen bonding, hydrophobic, ionic, electrostatic, van der Waals, π-π stacking) that take place in the solid state or in solution are generally key interactions for the foreseen applications. Therefore, in this review, we want to emphasize this important property of arene ruthenium metalla-assemblies by showing examples dealing with second coordination sphere interactions and how this can be better integrated in the design of these versatile supramolecular metal-based entities.

## Introduction

It is now well-known that the second coordination sphere (second shell) plays a major role in metal-based enzymatic transformations (Dudev et al., 2003). These additional interactions that take place in the proximity of the catalytic pocket can either stabilize the metal-substrate complex, stabilize the electronic state of the metal, orient the ligands to enhance the reactivity, act as proton and/or electron mediators, and so on (Ando et al., 1996; Botta, 2000; Steed, 2001; Haviv et al., 2018). More generally, these weak interactions (coordination, ionic, hydrogen bonding, hydrophobic, electrostatic, van der Waals, π-π stacking) are not only extremely important for biological processes, they are also a pillar of supramolecular chemistry. They allow the formation in the solid state of molecular networks (Hosseini, 2003), the preparation of liquid crystalline materials (Kato et al., 2018), the construction of coordination-driven assemblies (Fujita et al., 2005), as well as the generation of many other supramolecular systems (Wu et al., 2008; Zhou et al., 2017).

For many years, we have been involved in the field of coordination-driven self-assembly, using piano-stool complexes (also called half-sandwich complexes) as building blocks, and especially arene ruthenium complexes as biological agents (Therrien and Furrer, 2014; Therrien, 2015). The piano-stool unit provides three coordination sites at 90° from each other for a strategic coordination of ligands on metals (Therrien, 2009), which allows the design of 2D and 3D entities (Cook et al., 2013; Singh et al., 2014; Therrien, 2018). These metalla-assemblies possess different functional groups, situated either at the periphery or at the core of the assembly. They have good stability in solution, showing no dynamic ligand exchange under ordinary conditions (Garci et al., 2014). They come in different sizes, with or without a cavity, and they can be positively charged, thus showing various properties and solubility. Among these properties, host-guest chemistry, biological activity, DNA interactions, recognition, ion binding, and others have been identified. Therefore, in this perspective review, the biological applications of arene ruthenium metalla-assemblies are discussed from the second coordination sphere point of view, to better emphasize the importance of weak interactions in diverse properties. Ultimately, it can provide to those working in the field a different angle to envision the next generation of metalla-assemblies in biomedical fields.

## Sensing

**Figure 1:**
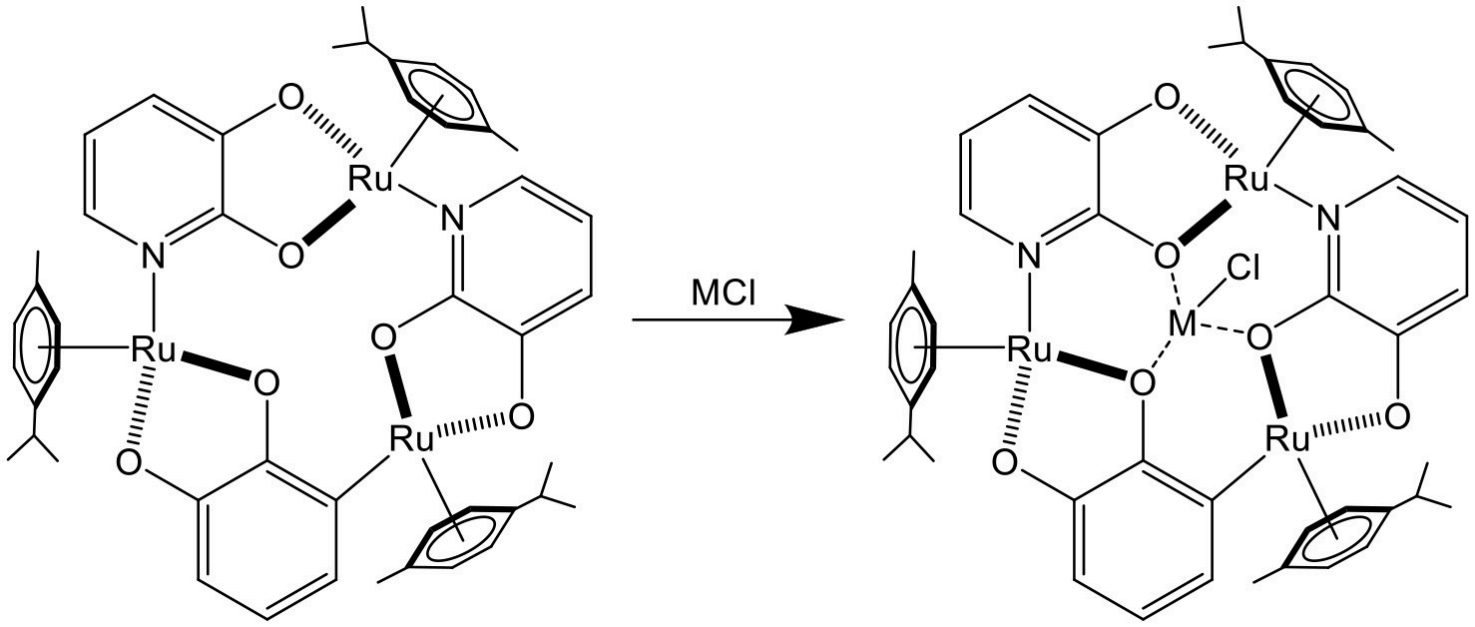
Sensing of MCI (M = Li, Na, K) in a trinuclear arene ruthenium metalla-cycle (Piotrowski et al., 2001).

The first example of arene ruthenium metalla-assemblies used for sensing was published in 2001 (Piotrowski et al., 2001). The electrochemical property of a trinuclear arene ruthenium metalla-cycle was exploited. Interestingly, upon the binding of alkali chloride salts in the triple-oxo binding site of the metalla-cycle (Figure 1), the oxidation potential of the trinuclear assembly was shifted by as much as 450 mV in the presence of LiCl, thus providing a redox-responsive signal upon guest binding.

**Figure 2:**
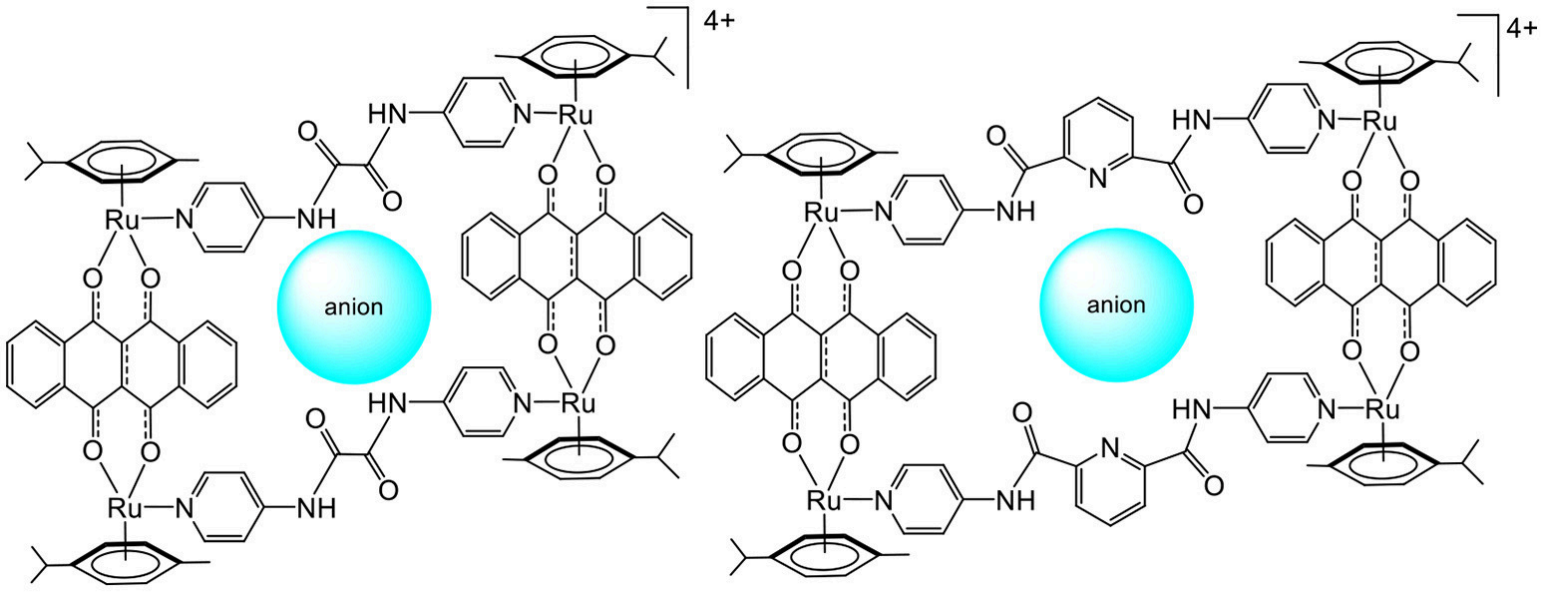
Sensing of polyanionic species in tetranuclear arene ruthenium metalla-assemblies (Vajpayee et al., 2011; Mishra et al., 2012).

Later, the possibility of using arene ruthenium metalla-assemblies for sensing biologically relevant substrates was explored by Chi and Stang (Vajpayee et al., 2011; Mishra et al., 2012). The cavity of arene ruthenium metalla-rectangles (Figure 2) has shown interactions with polyanionic compounds (oxalate, citrate, tartrate). The presence of multiple amido and pyridine groups within the core of the metalla-rectangles was crucial for the recognition process to take place. Moreover, the amido groups gave some structural flexibility to the systems, thus providing binding adaptability to optimize the interactions with anions. In these systems, the size of the cavity and the capacity to form several hydrogen-bonds with anions are crucial elements for the sensing process to take place.

These are simple examples in which the nature of the functional groups incorporated in the building blocks of the arene ruthenium metalla-assemblies has provided valuable second sphere coordination interactions to develop metalla-assembled sensors.

## DNA Interactions

**Figure 3:**
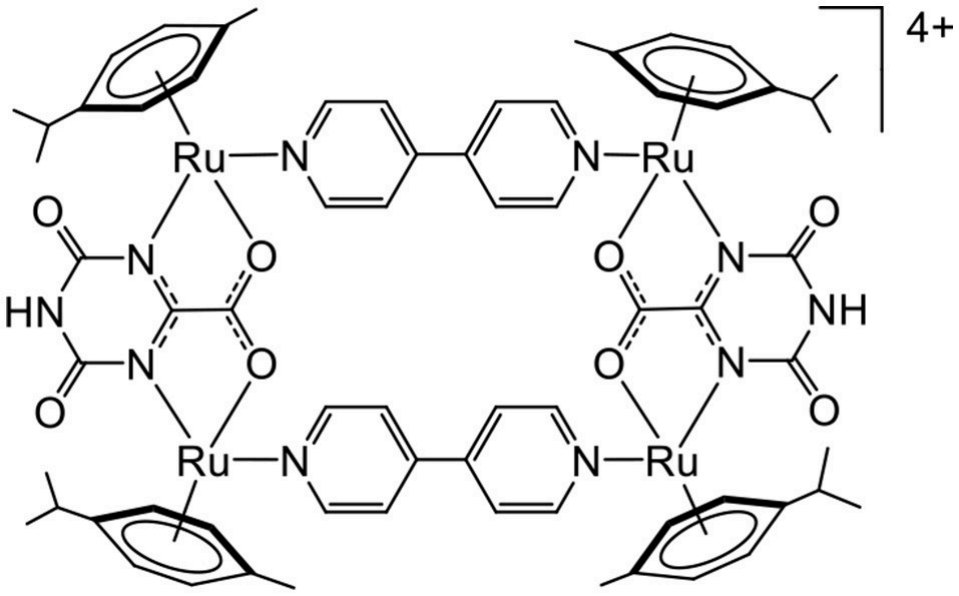
Arene ruthenium metalla-rectangle interacting with DNA (Linares et al., 2009).

The good water solubility and the presence of positive charges on most arene ruthenium metalla-assemblies are both advantageous properties for interactions with biomolecules. The first study dealing with arene ruthenium metalla-assemblies and DNA interactions was published in 2009 (Figure 3). The tetracationic bowl-shaped rectangle showed good interaction with calf-thymus DNA (Linares et al., 2009). Binding assays have suggested that the interactions take place in the major groove of the duplex DNA strand. Conformational changes in the DNA strand are probably due to electrostatic interactions between the cationic metalla-rectangle and the negatively charged surface of DNA as well as the good match between the size of the rectangle and the size of the major groove.

Following this initial study, several other arene ruthenium metalla-assemblies were tested as duplex DNA binders (Linares et al., 2010; Paul et al., 2015; Gupta et al., 2016, 2017). However, in the human genome, DNA sequences can fold into other thermodynamically stable structures such as hairpins and quadruplexes. These kinds of secondary structures are interesting targets for therapeutic applications (Zhao et al., 2010). Consequently, arene ruthenium metalla-assemblies have been used, for example, as quadruplex DNA stabilizers (Barry et al., 2009).

**Figure 4:**
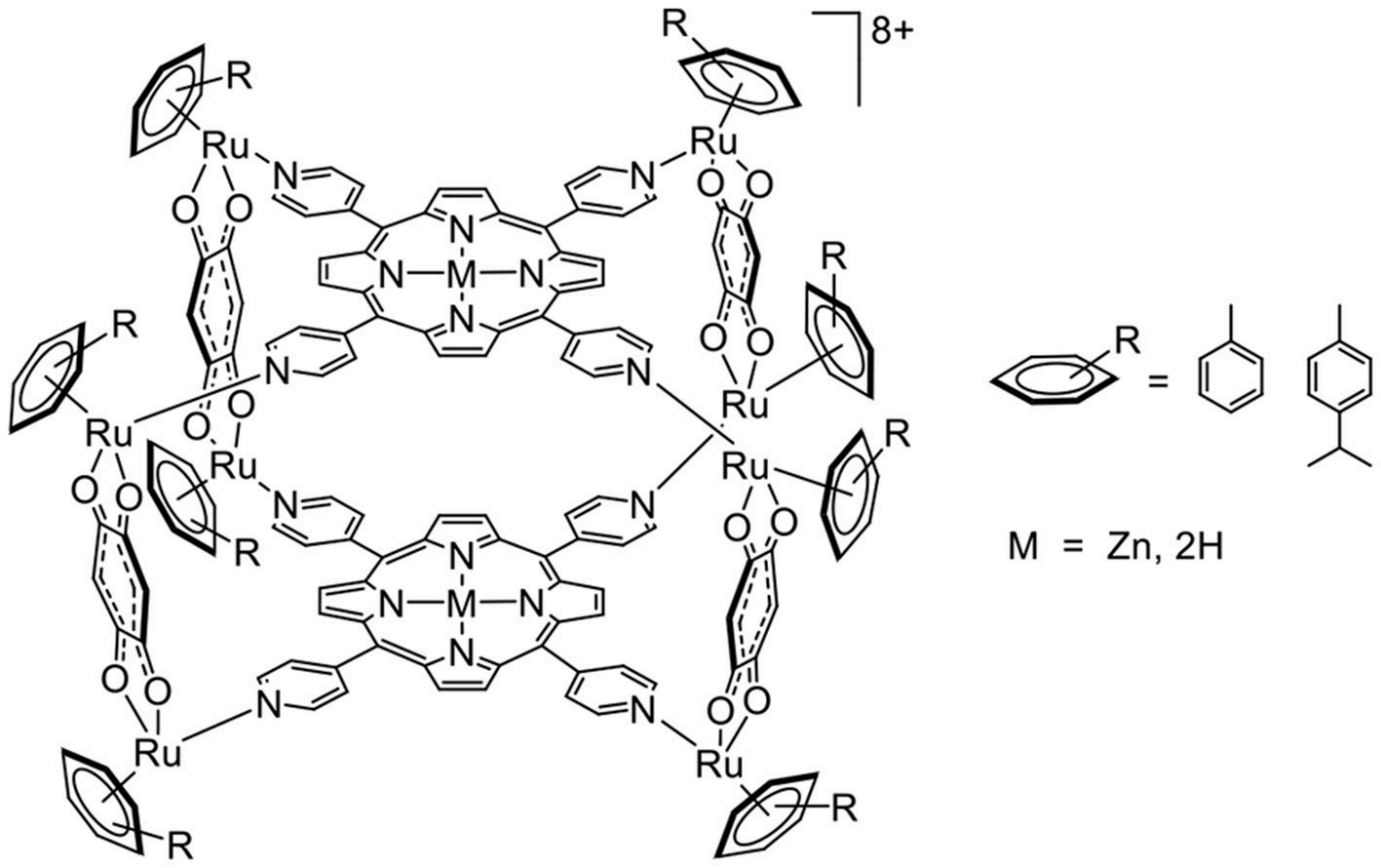
Arene ruthenium metalla-cubes interacting with G-quadruplexes (Barry et al., 2009).

A G-quadruplex is composed of guanine tetrads intercalated by cations, and it possesses a planar aromatic surface (Balasubramanian and Neidle, 2009). Therefore, cationic molecules with π-stacking affinity can potentially interact with G-quadruplexes. This possibility of interaction and stabilization was confirmed from the porphyrin-based arene ruthenium metalla-cubes (Figure 4), which showed good stabilization of telomeric and *c-myc* DNA quadruplexes. In such systems, we can assume that a combination of electrostatic and π-stacking (hydrophobic) interactions generates second coordination sphere interactions.

## Protein Interactions

Like DNA, proteins can positively interact with cationic compounds, and accordingly with positively charged arene ruthenium metalla-assemblies. Different proteins interacting with such metalla-assemblies have been identified. Interestingly, these studies have shown that metalla-assemblies of different shapes, sizes, structures and numbers of charges are able to interact with proteins, confirming that interaction with biomolecules is a common feature of arene ruthenium metalla-assemblies (Dubey et al., 2015; Elumalai et al., 2016, 2017).

**Figure 5:**
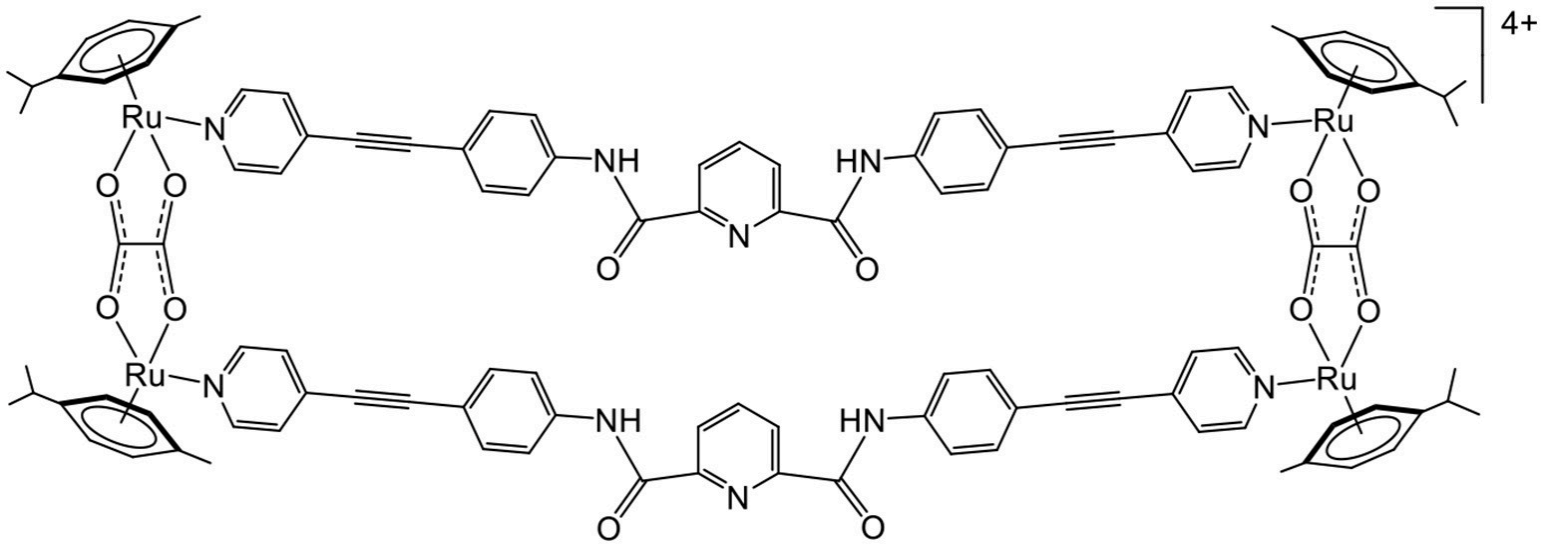
Arene ruthenium metalla-rectangle interacting with EGF protein (Mishra et al., 2014).

Indeed, an arene ruthenium metalla-rectangle with bis-amido pyridine containing linkers (Figure 5) shows strong interaction with enhanced green fluorescence protein (EGFP) (Mishra et al., 2014). Similarly, an arene ruthenium metalla-prism can disrupt the folded structures of albumin, transferrin, cytochrome-c, and other proteins (Paul et al., 2018), showing a great diversity of protein interactions. These examples suggest that the interactions between metalla-assemblies and proteins are mainly electrostatic. Therefore, to optimize the metalla-assembly protein interactions, and to gain a degree of selectivity for a specific protein, one cannot rely purely on electrostatic, hydrophobic, size-dependent or hydrogen-bond interactions. Only the multiplicity of second coordination sphere interactions can generate selective protein binders, which increases the complexity of designing arene ruthenium metalla-assemblies.

## Decomplexation Upon Interactions

**Figure 6:**
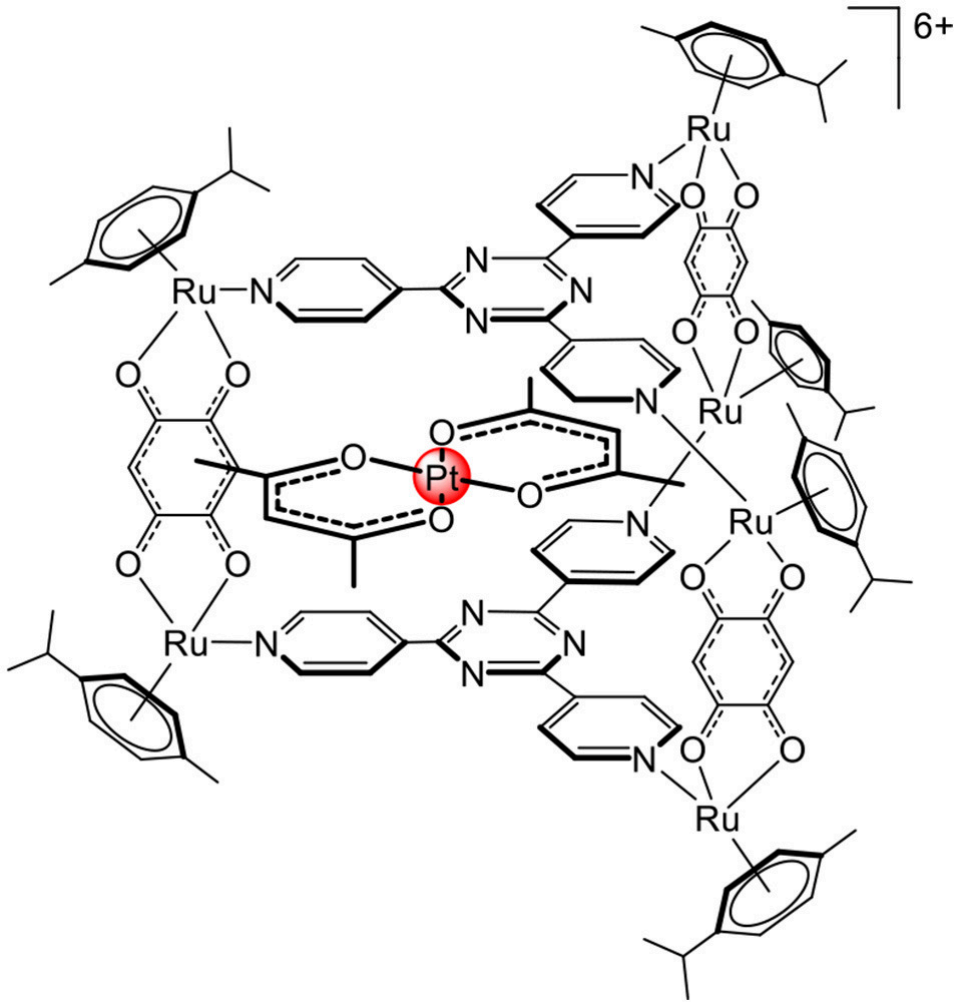
Complex –in-a-complex arene ruthenium metalla-prism (Therrien et al., 2008).

The first biological application of arene ruthenium metalla-assemblies was confirmed by the complex-in-a-complex system (Figure 6), in which a water-soluble arene ruthenium metalla-prism was used to transport the platinum acetylacetonate complex to cells (Therrien et al., 2008). Later, it was demonstrated that, after internalization, the guest complex was released, most likely upon disassembly of the cage compound (Mattsson et al., 2010). Furthermore, the metalla-prism can interact with biomolecules such as arginine, cysteine, glutathione, lysine, histidine (Paul et al., 2012a,b), and biomolecules possessing coordinating functional groups, thus being able to initiate the breakage of the cage compound. This suggests that despite being relatively robust to ligand exchange processes (Garci et al., 2014), second coordination sphere interactions can be used to disassemble arene ruthenium metalla-assemblies.

## Oxygen Interactions

In traditional photodynamic therapy (PDT), a photosensitizer interacts with oxygen to produce reactive oxygen species (ROS), and these ROS trigger cell death upon light activation (Patrice et al., 2003). Therefore, to evaluate the potential of using arene ruthenium metalla-assemblies as PDT agents, several metalla-assemblies coupled to photosensitizers were prepared and tested (Schmitt et al., 2009). The arene ruthenium units can modulate the solubility of the photosensitizers (Schmitt et al., 2008) as well as modify its photochemical behavior. Moreover, the cavity of arene ruthenium cages can transport and protect the photosensitizer (Schmitt et al., 2012).

**Figure 7:**
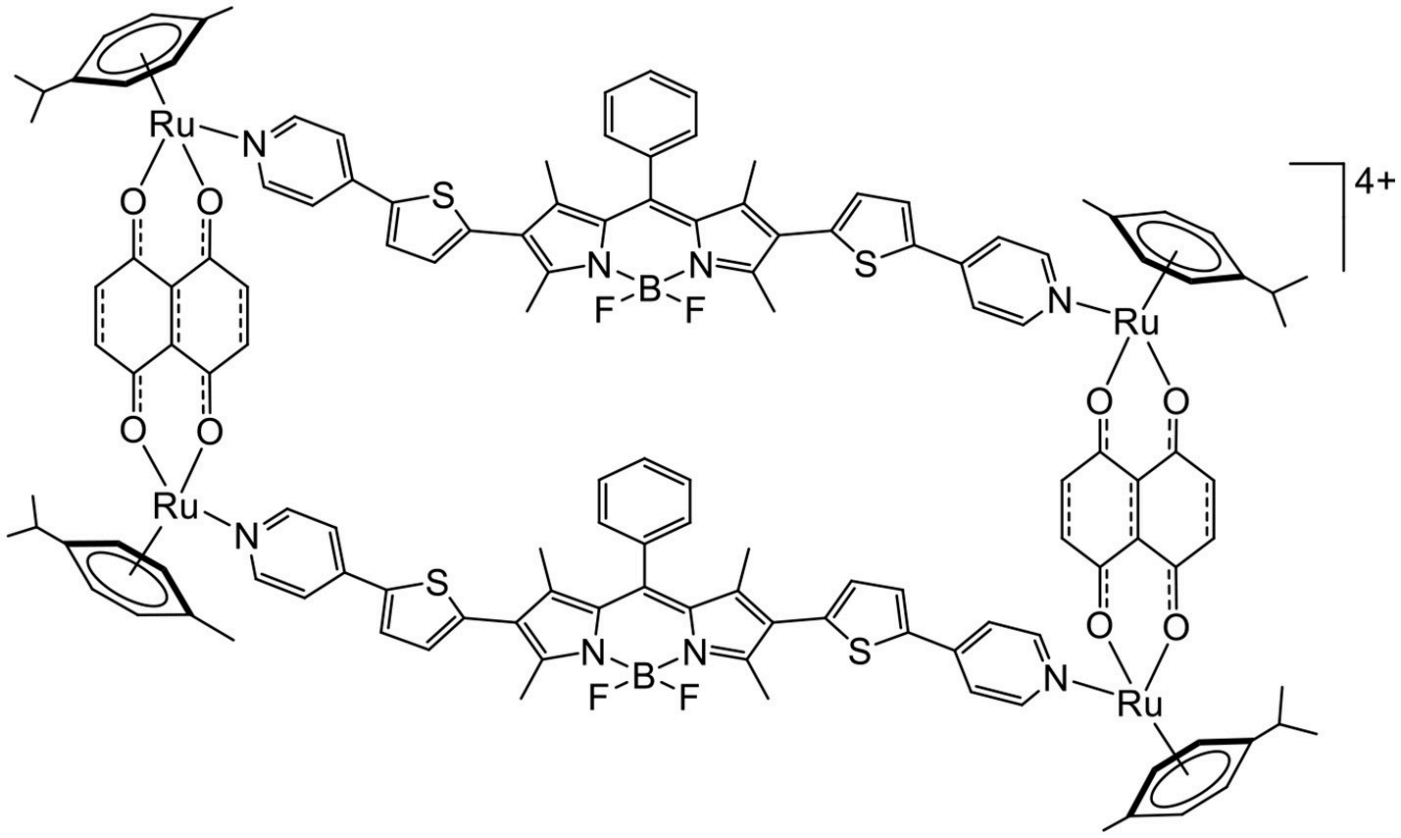
BODIPY-based tetranuclear arene ruthenium metalla-assemblies as potential PDT agent (Gupta et al., 2017).

Nevertheless, other types of molecules can interact with oxygen to generate ROS. This is the case of BODIPY, an interesting fluorescent dye with a high-quantum yield that can promote the production of ROS. Therefore, BODIPY-based linkers have been recently inserted in arene ruthenium metalla-rectangles (Figure 7), and the cytotoxicity of the compounds on various cancer cell lines was confirmed (Gupta et al., 2017). The fluorescence associated with the BODIPY units was exploited to localize the metalla-rectangle in the cytoplasm of cancer cells. However, the possibility of using such metalla-rectangles as PDT agents remains to be explored.

**Figure 8:**
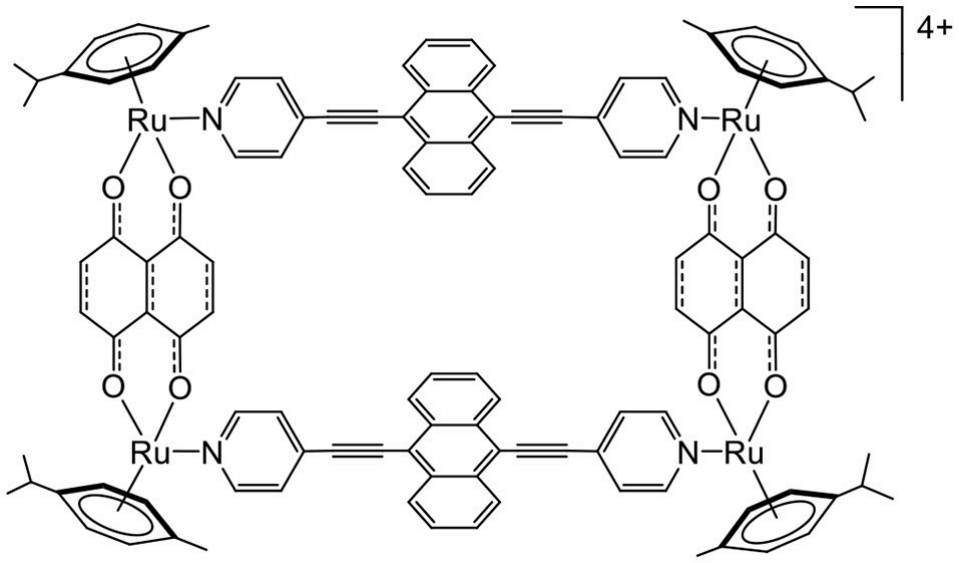
Anthracene-based tetranuclear arene ruthenium metalla-assemblies for o_2_ interactions (Gaschard et al., 2018).

Anthracene is another molecule that can react with oxygen (Aubry et al., 2003). Anthracene forms in the presence of oxygen and light activation, an endoperoxide intermediate. The endoperoxide formation is reversible, and oxygen can be released in a different environment. Therefore, knowing that arene ruthenium metalla-assemblies can be internalized to cells and can be coupled to photosensitizers, we have recently synthesized an anthracene-based metalla-rectangle (Figure 8) (Gaschard et al., 2018). Despite an unsuccessful endoperoxide formation on the metalla-rectangle, the ultimate goal of this project was to transport oxygen and a photosensitizer to cells for an optimization of PDT treatments in hypoxic cancers.

## Conclusion

As pointed out in this article, the second coordination sphere plays a major role in most biological and biomedical applications involving arene ruthenium metalla-assemblies. Therefore, the introduction of functional groups that can generate weak interactions with biomolecules on either the arene, the building blocks or the guest molecules is essential for the development of biologically active arene ruthenium metalla-assemblies. In the future, designing metalla-assemblies with second coordination sphere interactions in mind will be challenging, but it could provide the next generation of arene ruthenium derivatives for biological and biomedical applications.

## Author Contributions

The author confirms being the sole contributor of this work and has approved it for publication.

### Conflict of Interest Statement

The author declares that the research was conducted in the absence of any commercial or financial relationships that could be construed as a potential conflict of interest.
